# Arthritis in adults with community-acquired bacterial meningitis: a prospective cohort study

**DOI:** 10.1186/1471-2334-6-64

**Published:** 2006-03-29

**Authors:** Martijn Weisfelt, Diederik van de Beek, Lodewijk Spanjaard, Jan de Gans

**Affiliations:** 1 Department of Neurology, Centre of Infection and Immunity Amsterdam (CINIMA), Academic Medical Centre, University of Amsterdam, Amsterdam, The Netherlands; 2 Medical Microbiology, Centre of Infection and Immunity Amsterdam (CINIMA), Academic Medical Centre, University of Amsterdam, Amsterdam, The Netherlands

## Abstract

**Background:**

Although the coexistence of bacterial meningitis and arthritis has been noted in several studies, it remains unclear how often both conditions occur simultaneously.

**Methods:**

We evaluated the presence of arthritis in a prospective nationwide cohort of 696 episodes of community-acquired bacterial meningitis, confirmed by culture of cerebrospinal fluid, which occurred in patients aged >16 years. The diagnosis of arthritis was based upon the judgment of the treating physician. To identify differences between groups Fisher exact statistics and the Mann-Whitney U test were used.

**Results:**

Arthritis was recorded in 48 of 696 (7%) episodes of community-acquired bacterial meningitis in adults. Joint-fluid aspirations were performed in 23 of 48 patients (48%) and joint-fluid cultures yielded bacteria in 6 of 23 patients (26%). Arthritis occurred most frequently in patients with meningococcal meningitis (12%). Of the 48 patients with bacterial meningitis and coexisting arthritis, four died (8%) and 10 (23%) had residual joint symptoms.

**Conclusion:**

Arthritis is a common manifestation in patients with community-acquired bacterial meningitis. Functional outcome of arthritis in bacterial meningitis is generally good because meningococcal arthritis is usually immune-mediated, and pneumococcal arthritis is generally less deforming than staphylococcal arthritis. Nevertheless, additional therapeutic measures should be considered if clinical course is complicated by arthritis. In patients with infectious arthritis prolonged antibiotic therapy is mandatory.

## Background

The clinical spectrum of complications in patients with bacterial meningitis is broad. Although the coexistence of bacterial meningitis and arthritis has been described in several studies, the prevalence, patient characteristics, and outcome of patients with arthritis in bacterial meningitis are unknown [1-4]. Therefore, we assessed the prevalence and clinical course of arthritis in a prospective cohort of 696 episodes of adulthood bacterial meningitis.

## Methods

The Dutch Meningitis Cohort Study, a prospective nationwide observational cohort study in the Netherlands, includes 696 episodes of community-acquired acute bacterial meningitis [5]. Inclusion and exclusion criteria are described more extensively elsewhere [5]. In summary, all patients were aged >16 years and had cerebrospinal fluid (CSF) culture proven bacterial meningitis. CSF culture yielded *Streptococcus pneumoniae *in 352 episodes (51%), *Neisseria meningitidis *in 257 episodes (37%), *Staphylococcus aureus *in 9 episodes (1%) and other bacteria in 78 episodes (11%) [5]. The Dutch Meningitis Cohort Study was approved by our ethics committee and informed consent was obtained from all participating patients or their legally authorised representatives. Information, including explicit queries about arthritis, was collected by means of a case record form. The diagnosis of arthritis was based upon the judgment of the treating physician and was based on clinical grounds with or without additional laboratory examinations (*e.g*., joint-fluid aspirations). Discharge letters were collected from all patients in whom arthritis was recorded. Arthritis was considered of early-onset if symptoms occurred before or within 4 days after admission for bacterial meningitis. Predisposing factors for bacterial arthritis were predefined as age ≥ 80 years, immunodeficiency (*e.g*., diabetes mellitus), preexisting joint disease (*e.g*., rheumatoid arthritis), and prosthetic joints [6,7]. To identify differences between groups Fisher exact statistics and the Mann-Whitney U test were used.

## Results

Arthritis was recorded in 48 of 696 episodes of bacterial meningitis (7%), occurring in 48 patients. Characteristics of these patients are listed in Table 1. CSF cultures revealed *N. meningitidis *in 32 of 48 patients (67%), *S. pneumoniae *in 11 (23%), and other bacterial species in 5 patients (10%). The incidence of arthritis was higher in patients with meningococcal meningitis than in those with meningitis due to other bacterial species (32 of 257 patients [12%] vs. 16 of 439 patients [4%]; P < .001). At least one predisposing factor for bacterial arthritis (definition see above) was present in 11 of 48 patients (23%). The proportion of patients with predisposing factors was higher among patients with pneumococcal meningitis, compared with those with meningococcal meningitis (7 of 11 [78%] vs. 2 of 32 [6%]; P < .001).

**Table 1:** Characteristics of 48 patients with community-acquiredbacterial meningitis and coexisting arthritis*

**Characteristics**	**Patients (n = 48)**
Sex, no. male/no. female	21/27
Age, median years (range)	49 (17–90)
Predisposing factors for bacterial arthritis	
Immunodeficiency†	6 (13)
Preexisting joint pathology‡	3 (6)
Age ≥ 80 years	4 (8)
Blood culture positive	29/46 (63)
Erythrocyte sedimentation rate – mm/hour, median (range)	45 (5 – 289)
Blood leukocyte count – 10^9^/L, median (range)	18 (4 – 40)
Results of cerebrospinal fluid culture	
*Neisseria meningitidis*	32 (67)
*Streptococcus pneumoniae*	11 (23)
Other bacteria§	5 (10)
Positive synovial fluid culture	6/23 (26)
Arthritis present ≤ 4 days after admission	29 (60)
Arthritis present on presentation	15 (31)
Time between admission and arthritis, median days (range)	5 (-2 – 12)
Monoarticular arthritis	29 (60)
Knee	14/29 (48)
Wrist	5/29 (17)
Ankle	4/29 (14)
Other¶	6/29 (21)
Polyarticular arthritis	19 (40)
Knee	15/19 (79)
Residual joint symptoms in surviving patients$	10/44 (23)

*Data are given as no. of patients with characteristic/no. evaluated (%), unless otherwise indicated.

†alcoholism (n = 1), diabetes mellitus (n = 4) and splenectomy (n = 1).

‡rheumatoid arthritis (n = 1), gout (n = 1), hip prostheses (n = 1).

§ *Haemophilus influenzae *(n = 2), *Escherichia coli *(n = 1), group B streptococcus (n = 1) and group G streptococcus (n = 1).

¶ hip (n = 2), shoulder (n = 2), elbow (n = 2).

$cause of meningitis: *N. meningitidis *(n = 5), *S. pneumoniae *(n = 4), group B streptococcus (n = 1).

Arthritis was of early-onset in 29 of 48 patients (60%) and was already present at presentation in 15 patients (31%). The median time between admission and the first symptoms of arthritis was 5 days (range, 2 days before to 12 days after admission). Arthritis was significantly more often of early-onset in patients with pneumococcal meningitis than in those with meningococcal meningitis (10 of 11 [91%] vs. 17 of 32 [53%]; P = .03). Overall, the majority of patients (60%) had monoarticular involvement; 10 of 11 patients with pneumococcal meningitis (91%) vs. 16 of 32 patients with meningococcal meningitis (50%; P = .03). Large joints were involved in all patients and additional involvement of small joints was recorded in 3 patients. The knee was the most frequently involved joint, both in patients with monoarthritis (14 of 29 [48%]) and in those with polyarthritis (15 of 19 [79%]).

Joint-fluid aspirations were performed in 23 of 48 patients (48%), of which 19 (83%) were performed after the initiation of antibiotic therapy. The median duration between admission and joint-fluid aspiration was 7 days (range, 0–28 days). Joint-fluid cultures yielded bacteria in 6 of 23 patients (26%): *N. meningitidis *in 5 patients, *S. pneumoniae *in 1. Bacterial species cultured from joint-fluid and CSF in these 6 patients were identical. All 6 patients with positive joint-fluid cultures had early-onset arthritis and all underwent joint-fluid aspirations within 3 days of initiation of antibiotic therapy. In 17 patients with negative cultures joint-fluid was purulent in 8 patients (47%).

Outcome was recorded for all 48 patients. The mortality rate in patients with arthritis was lower than in those without arthritis (4 of 48 [8%] *vs*. 139 of 648 [21%]; P = .03). In patients with pneumococcal meningitis the mortality rate was similar in those with and without arthritis (3 of 8 [27%] *vs*. 104 of 341 [30%]; P = 1.0). None of the 32 patients with meningococcal meningitis and arthritis died as compared to 19 of 225 without arthritis [8%; P = 0.14). Patients who died were significantly older than those surviving meningitis (median 75 years [range, 60 to 90 years] vs. 47 years [17 to 84 years]; P = .01). Physical examination at discharge revealed a limited range of motion for the involved joint(s) in 10 of 44 surviving patients (23%); 4 of 7 surviving patients with pneumococcal meningitis (58%) and 5 of 32 survivors with meningococcal meningitis (16%) (P = .04). These limitations were without significant disability in 9 patients, only one patient had severe joint destruction. There was no association between the presence of residual joint symptoms and predisposing factors for arthritis, positive blood culture, an elevated blood leukocyte count or any other inflammatory parameter. The median duration of intravenous antibiotic therapy in surviving patients was 13 days (range, 8–43 days). In 4 patients parenteral antibiotic therapy was followed by oral antibiotics (median duration 26 days, range 18–42 days).

## Discussion

Our study shows that arthritis is a common manifestation in patients with community-acquired bacterial meningitis. Overall, arthritis was diagnosed in 7% of patients. For patients with meningococcal meningitis this percentage was even higher (12%). This is the first large prospective study that specifically evaluates the presence of arthritis in adults with bacterial meningitis. A previous prospective case series in adults with bacterial meningitis showed a much lower prevalence of arthritis (2%) [1]. However, this study was relatively small in sample size (86 patients) and only 6% of included patients had meningococcal meningitis. In retrospective cohort studies on meningococcal meningitis, arthritis was estimated to occur in 5 to 11% of patients [8,9]. In a study on 1158 bacterial isolates of patients with joint infections, the most common causative organisms in adults were *S. aureus *and *S. pneumoniae *[10]. In a study on patients with pneumococcal septic arthritis, meningitis was noted as the underlying extra-articular focus of infection in 16 of 105 adults (15%) [2].

In the present study, cultures of joint-fluid yielded bacteria in only 26% of the joint-fluid specimens. Synovial fluid cultures are estimated to be positive in approximately 90% of the cases of non-gonococcal bacterial arthritis, if performed before the initiation of antibiotic therapy [6]. Microbiological yield is much lower if specimens are collected after the administration of antibiotics [6,11]. In our study, 90% of the joint-fluid aspirations were performed in patients who had already received antibiotics. Therefore, the low rate of positive synovial fluid cultures probably underestimates the proportion of patients with an infectious aetiology. Other diagnostic considerations in these patients are gout and pseudogout, which commonly flare up during stress and acute medical illness.

Although history and physical examination may provide important clues, it is often difficult to discriminate infectious from non-infectious causes of joint inflammation [6,11]. The observed pattern of affected joints in our study (predominantly monoarticular with involvement of large joints) does not differentiate between infectious and non-infectious arthritis [4,8,12]. Multiple joint involvement does not necessarily imply a non-infectious aetiology of the arthritis, as it occurs in 10% of patients with infectious arthritis [12]. Treatment of septic arthritis consists of joint drainage and antibiotic therapy. Delay in initiation of appropriate therapy may lead to irreversible loss of joint function [2,6,12]. Depending on the bacterial pathogen, the recommended duration of intravenous antibiotic therapy for septic arthritis is 2–4 weeks, often followed by oral antibiotics [6,12]. The common treatment of bacterial meningitis consists of 7–14 days intravenous antibiotics, so modification of the antimicrobial regimen in patients with bacterial meningitis should be considered if clinical course is complicated by arthritis [5].

Although some limitation in the range of movement was found in 23% of patients with meningitis and arthritis, functional outcome was good in the majority of surviving patients. This relatively benign clinical course is in contrast with previous studies on septic arthritis, which described severe irreversible loss of joint function in 25–50% of patients [11]. This difference in outcome might be caused by the much lower incidence of infections with *S. aureus *in our study, compared with these studies on septic arthritis [11]. Infection with *S. aureus *has been associated with severe joint destruction [11]. In our study, almost all cases with arthritis occurred in patients with meningitis due to *S. pneumoniae *or *N. meningitidis*, the most common causative species of community acquired bacterial meningitis [5]. The relatively benign outcome of arthritis is concordant with previous reports in literature on pneumococcal and meningococcal arthritis [2,8].

Early-onset arthritis and monoarticular involvement were more common in patients with pneumococcal meningitis than in patients with meningococcal infections. These findings suggest that in pneumococcal meningitis arthritis is predominantly a bacterial infection, whereas in meningococcal meningitis it is more often immunomediated [3,4,11]. In immunomediated arthritis, the host's immunologic response to the bacterium leads to immune-complex deposits within the joint [3]. Patients with immunomediated arthritis during meningococcal infection, typically develop complaints from day 5 of illness, during recovery from the infection, which usually involve the great joints [8,9].

The most important limitation of our study is the lack of strict criteria for the diagnosis of arthritis. Synovial fluid was not routinely analyzed and in most patients joint-fluid aspiration was performed after the initiation of antibiotic therapy. In addition, we did not routinely collect information about additional treatment strategies such as surgical procedures and physical therapy. We only included patients with community-acquired bacterial meningitis and excluded those with a neurosurgical device or recent surgery [5]. Bacterial meningitis due to *S. aureus *is most commonly hospital-acquired, and staphylococcal arthritis is especially associated with residual symptoms [5].

## Conclusion

In conclusion, our study shows that arthritis is a common manifestation in patients with community-acquired bacterial meningitis. Functional outcome of arthritis in bacterial meningitis is generally good because meningococcal arthritis is usually immune-mediated, and pneumococcal arthritis is generally less deforming than staphylococcal arthritis. Nevertheless, additional therapeutic measures should be considered if clinical course is complicated by arthritis. In patients with infectious arthritis prolonged antibiotic therapy is mandatory.

## Competing interests

The author(s) declare that they have no competing interests.

## Authors' contributions

MW participated in the design of the study, performed the statistical analysis, and participated in the writing of the article. DvdB and JdG participated in the design and coordination of the study and participated in the writing of the article. LS participated in the writing of the article. All authors read and approved the final manuscript.

## Pre-publication history

The pre-publication history for this paper can be accessed here:


